# Comprehensive molecular characterization of complete mitogenome assemblies of 33 *Eimeria* isolates infecting domestic chickens

**DOI:** 10.1186/s13071-023-05712-5

**Published:** 2023-03-19

**Authors:** Xuan Zhou, Lidan Wang, Pengchen Zhu, Zijiang Yang, Zhao Wang, Yijun Chen, Xiaobin Gu, Ran He, Jing Xu, Bo Jing, Guangyou Yang, Shun Chen, Shuangyang Wu, Yue Xie

**Affiliations:** 1 Department of Parasitology, College of Veterinary Medicine, Sichuan Agricultural University, Sichuan, 611130 China; 2 Tokyo University of Marine Science and Technology, Konan Minato-Ku, Tokyo, 1088477 Japan; 3 Institute of Preventive Veterinary Medicine, Sichuan Agricultural University, Sichuan, 611130 China; 4 Gregor Mendel Institute, Austrian Academy of Sciences, 1030 Vienna, Austria

**Keywords:** Chicken coccidia, Population genetics, Mitogenome, Phylomitogenomics, Genetic markers

## Abstract

**Background:**

Coccidiosis caused by *Eimeria* is one of the most severe chicken diseases and poses a great economic threat to the poultry industry. Understanding the evolutionary biology of chicken *Eimeria* parasites underpins development of new interactions toward the improved prevention and control of this poultry disease.

**Methods:**

We presented an evolutionary blueprint of chicken coccidia by genetically characterizing complete mitogenome assemblies of 33 isolates representing all seven known *Eimeria* species infecting chickens in China. Further genome- and gene-level phylogenies were also achieved to better understand the evolutionary relationships of these chicken *Eimeria* at the species level.

**Results:**

33 mitogenomes of chicken eimerian parasites ranged from 6148 bp to 6480 bp in size and encoded typical mitochondrial compositions of apicomplexan parasites including three protein-coding genes (PCGs), seven fragmented small subunit (SSU) and 12/13 fragmented large subunit (LSU) rRNAs. Comparative genomics provided an evolutionary scenario for the genetic diversity of PCGs-cytochrome c oxidase subunits 1 and 3 (*cox*1 and *cox*3) and cytochrome b (*cyt*b); all were under purifying selection with *cox*1 and *cox*3 being the lowest and highest evolutionary rates, respectively. Genome-wide phylogenies classified the 33 *Eimeria* isolates into seven subgroups, and furthermore *Eimeria tenella* and *Eimeria necatrix* were determined to be more closely related to each other than to the other eight congenic species. Single/concatenated mitochondrial protein gene-based phylogenies supported *cox*1 as the genetic marker for evolutionary and phylogenetic studies for avain coccidia.

**Conclusions:**

To our knowledge, these are the first population-level mitogenomic data on the genus *Eimeria*, and its comprehensive molecular characterization provides valuable resources for systematic, population genetic and evolutionary biological studies of apicomplexan parasites in poultry.

**Graphical Abstract:**

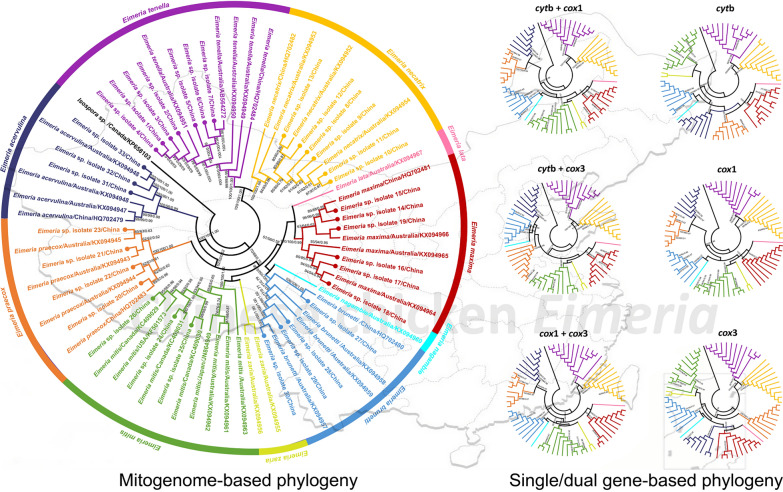

**Supplementary Information:**

The online version contains supplementary material available at 10.1186/s13071-023-05712-5.

## Background

Chicken coccidiosis, caused by apicomplexan parasites of the genus *Eimeria*, is a widespread and highly pathogenic avian disease posing a significant threat to the poultry industry worldwide [1, 2]. There are seven species of *Eimeria* responsible for morbidity and socioeconomic burdens including *Eimeria necatrix*, *E. tenella*, *E. maxima*, *E. praecox*, *E. mitis*, *E. brunetti* and *E. acervulina* [3]. Besides, three cryptic *Eimeria* operational taxonomic units (OTUs) were recently identified as endemic to Australian chicken populations and later given the names *Eimeria lata*, *E. nagambie* and *E. zaria* based on their genotypic and phenotypic properties [4–6]. Infections with *Eimeria* spp. can damage the digestive tract of chickens and lead to malabsorption of nutrients and lethal diarrhea, which is reflected in clinical symptoms: poor weight gain, low egg production and even death [1, 3]. There are many reports of coccidiosis prevalence in countries worldwide, with 70–92% prevalence in Romania (92%), Greece (86%), Brazil (91%), India (81%), Algeria (72%), Turkey (70%) and China (88%), and multi-*Eimeria* species infections predominate [7–14]. The annual economic loss for chicken farmers has been estimated at over £10.4 billion worldwide [15].

Because of similar oocyst morphotypes and overlapping biological features of chicken *Eimeria* spp. [16, 17], species identification based on morphological characteristics is difficult. Therefore, there is an urgent need to develop an efficient approach to identify *Eimeria* infections for clinical diagnosis; moreover, phylogenetic relationships and biogeographic range evolution of diverse strains can also provide valuable information to support diagnosis and control of chicken coccidiosis. Therefore, it might be possible to achieve this goal by utilizing next-generation sequencing (NGS) approach-based species identification, phylogenetic constructions and population genetic analyses. Encouragingly, Vermeulen and colleagues have established a ribosomal protein 18 (18S)-based NGS methodology to assess *Eimeria* communities of the Australian marsupial [18]. Hinsu et al. have also reported the application of Illumina MiSeq deep sequencing to 18S amplicons of chicken *Eimeria* and achieved detection of all seven validated species including their cryptic genotypes [19]. In addition, increased genetic evidence showed that the taxonomy and diversity of certain taxa or specific groups of chicken *Eimeria* can be equally revealed by using the mitochondrial (mt) gene-based NGS owing to matrilineal inheritance, absent recombination and rapid evolution rate of these molecules [20–22]. For example, Hauck et al. showed that using NGS to sequence mt cytochrome c oxidase subunit 1 (*cox*1) was efficient for identification of *Eimeria* species that circulated in chicken flocks [13]. Similarly, Snyder et al. also established the mt cytochrome c oxidase subunit 3 (*cox*3) amplicon library-based NGS pipeline analysis to differentiate *E. acervulina*, *E. tenella*, *E. maxima*, *E. mitis*, *E. necatrix* and *E. praecox* from mixed infected samples [23]. Nevertheless, it appears noteworthy that the single gene/locus-based NGS approach fails to provide sufficient genetic information to understand variations at the species and genus levels. Consequently, using NGS-based mitogenomic data becomes an ideal candidate strategy because it can not only provide comprehensive molecular insights into intra-/interspecific variability but also yields a broad taxonomy-range phylogenetic and evolutionary justification for *Eimeria* parasites [24, 25].

Herein, we sequenced complete mitogenomes of 33 *Eimeria* isolates infecting domestic chickens in China and genetically characterized these isolates by comparisons with other chicken eimerian parasites for which mitogenomic datasets are available in public databases. Furthermore, genome- and gene-level phylogenetic analyses were achieved to better understand the evolutionary relationships of chicken *Eimeria* at the species level. This comprehensive molecular information will provide valuable resources for systematic, population genetic and evolutionary biological studies of *Eimeria* parasites in poultry.

## Methods

### Litter sampling, parasite analysis and DNA extraction

From 2019 to 2021, 153 commercial farms that were in the finishing phase of the productive cycle were selected and sampled as part of ongoing surveys of coccidia on commercial broiler farms in China: 32 in Sichuan, 30 in Henan, 25 in Anhui, 23 in Chongqing, 18 in Guangdong, 15 in Fujian and 10 in Zhejiang. Litter samples were randomly collected from each shed while walking in a “zigzag” pattern [26]. About 250-g litter from each sampling was bagged and transported to the laboratory where it was kept at 4 °C. Oocysts per gram (OPG) of each litter was quantitated using a McMaster chamber. For species identification, positive litter samples were mashed and suspended with running water, followed by filtration through a series of sieves (212, 180, 75 and 45 mm; Endecotts, London, UK) and culture in a 5% (w/v) potassium dichromate solution for 72 h at 28 °C under forced aeration. Oocysts were sporulated and microscopically speciated using morphometric keys [27, 28]. Furthermore, pure sporulated oocysts from 33 isolates that represent all known *Eimeria* species infecting chickens were enriched with saturated sodium nitrate flotation technique. One hundred sporulated oocysts from each isolate were used for genomic DNA extraction. After vortex breakdown, the total genomic DNA was extracted from oocysts using the Genomic DNA Kit (TIANGEN, Beijing, China). Following the PCR amplification using *Eimeria*-specific primers ERIB1 (forward: 5′-ACCTGGTTGATCCTGCCAG-3′) and ERIB10 (reverse: 5′-CTTCCGCAGGTTCACCTACGG-3′) [29] and comparing with the targeted 18S regions, those isolates were determined to share > 99.5% identities with query sequences in GenBank nos. KT184333 (*E. acervulina*), KT184337 (*E. brunetti*), KT184346 (*E. maxima*), KT184349 (*E. necatrix*), KT184351 (*E. praecox*), KT184354 (*E. tenella*) and FR775307 (*E. mitis*).

### Genome sequencing, assembly and annotation

**Fig. 1:**
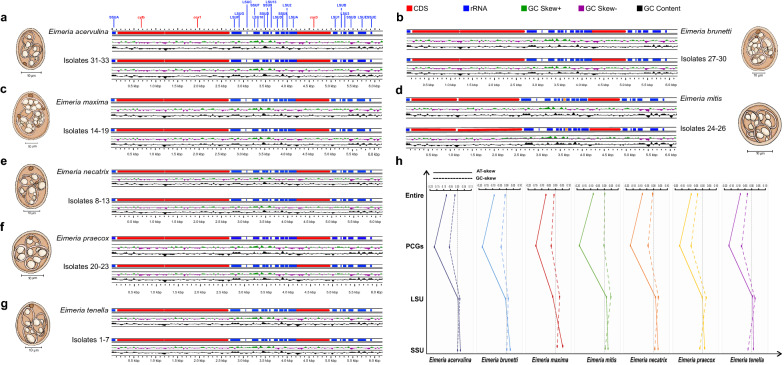
Structure views of mitogenomes of 33 chicken *Eimeria* isolates identified in this study. **a–g** Mitochondrial genome organizations of the *Eimeria* isolates 1–33 corresponding to seven chicken-infecting *Eimeria* species. Isolates in the same species are arranged together and contrast with the reported mitogenome organization. Red color indicates protein-coding genes, blue color indicates fragments of rRNA genes, green and purple colors indicate positive and negative GC-skew, respectively, and black color indicates the GC content of every position in the genome. **h** The average AT-skew and GC-skew values of the complete mitogenomes (Entire), concatenated PCG datasets (PCGs), LSU and SSU rRNAs of each species

After species identification, approximately 2 million sporulated oocysts from each of the 33 isolates were used for mitogenome sequencing. Following quality and quantity assessment, ~ 3 µg high-quality DNA from each sample was fragmented to construct 350-bp paired-end (PE) libraries and sequenced on an Illumina HiSeq X-TEN platform (BerryGenomics, Beijing, China). The clean reads (~ 1.8 Gb) were assembled with MITObim [30] using the available mitogenomes of other *Eimeria* species from GenBank (GenBank nos. JN864949, KX094949, KX094954, HQ702479, HQ702480, HQ702481, HQ702483 and KC409031) as the references. These assembled mitogenomes were also validated by PCR amplifications using four overlapping fragments. These four overlapping fragments were located between cytochrome b (*cyt*b) and *cox*1 (~ 2.0 kb), between *cox*1 and large subunit A (LSUA) (~ 3.0 kb), between LSUA and large subunit E (LSUE) (~ 2.0 kb) and between LSUE and *cyt*b (~ 1.0 kb), respectively (Fig. 1), and their corresponding PCR primers were designed based on the aforementioned mitogenome sequences of *Eimeria* species and are shown in Additional file 1: Table S1. PCR reactions were achieved using a 50-μl reaction volume containing 3 μl genomic DNA (≥ 10 ng), 25 μl 2 × HiFi TransTaq PCR SuperMix (TaKaRa, Biotech, Dalian, China), 1 µl sense primer (10 pmol), 1 µl anti-sense primer (10 pmol) and 20 µl nuclease-free water. The PCR conditions were: 95 °C for 5 min denaturation, 95 °C for 45 s for 30 cycles and 45 ~ 55 °C for 45 s, and 68 °C for 1 ~ 3 min according to the Tm values and the product lengths, and a final extension at 68 °C for 10 min. After agarose gel detections, all target amplicons were column-purified and sequenced either directly or following sub-cloning into the pMD19-T vector (TaKaRa, Biotech, Dalian, China). Each amplicon was sequenced three times to ensure maximum accuracy. Mitogenome annotation was performed with MITOS [31] and manual inspection based on a whole genome-guided alignment using poultry *Eimeria* spp. for which complete mitogenome sequences were available so far.

### Sequence characterization

Nucleotide composition and codon usage of 33 eimerian mitogenomes were measured using Geneious and PhyloSuite [32, 33]. The nucleotide skewness of mitogenomes was performed using the formulas [34]: AT skew = (A − T)/(A + T) and GC skew = (G − C)/(G + C). Nucleotide and amino acid divergences of protein-coding genes (PCGs) were determined using DNAstar [35]. The multi-alignment of PCGs obtained from 33 sequenced eimerian isolates and available chicken eimerian parasites including newly recognized *E. lata*, *E. nagambie* and *E. zaria* were separately aligned using MEGA software [36]. Based on those alignments, the sliding window analysis was used to compute the nucleotide diversity Pi (π) using a 200-bp window and 20-bp steps, followed by assessment of genetic structure using the Wright’s fixation index (*F*_ST_) with 1000 replicates as a permutation test in DnaSP ver. 5.10 [37]. In addition, the evolutionary rate and the ratio of the nonsynonymous substitution (Ka) and synonymous substitution (Ks) of each PCG were calculated using KaKs_Calculator [38]. Genetic distances among these 10 *Eimeria* species mitogenomes were calculated based on Kimura-2-parameter (K2P) [39] with MEGA X.

### Phylogenetic characterization

The phylogenies were reconstructed based on the mitogenomic datasets of 33 *Eimeria* isolates and other related species (Additional file 2: Table S2). Nucleotide sequence alignments were yielded from the complete mitogenomes, concatenated datasets of dual/triple PCGs or individual PCG of coccidian parasites using MAFFT ver. 7.271 [40], followed by filtration of the ambiguous regions with GBLOCKS ver. 0.91b [41]. Three algorithms including the maximum parsimony (MP), maximum-likelihood (ML) and Bayesian inference (BI) were used to reconstruct the phylogenetic relationships of chicken *Eimeria* species, and *Isospora* sp. (GenBank no. KP658103) was treated as the outgroup and included in each analysis. For the MP analysis, the complete nucleotide sequence dataset of mitogenomes or multi-alignments of the nucleotide sequence for the single, dual or triple PCGs were analyzed using equally weighted parsimony and heuristic searches with a tree-bisection-reconnection (TBR) branch-swapping in PAUP* [42]. One thousand replicates of Wagner trees (using random addition sequences) were chosen, and five trees of each replication were saved, followed by obtaining the optimal topology with the Kishino-Hasegawa method. Bootstrap resampling with 1000 replications was computed for each nodal support. The ML analysis was implemented with PHYML ver. 3.0.1 [43] using the optimal evolutionary models “TIM + F + I + G4” for the complete mitogenomic dataset and “GTR + F + I + G4” for the dual and triple concatenated PCGs and individual PCG datasets that were selected with the “Auto” option on W-IQ-TREE web server (http://iqtree.cibiv.univie.ac.at) according to Bayesian information criterion (BIC). ML trees were reconstructed with a 10,000-replicate and an ultra-fast bootstrap approximation. Within the BI trees, the optimal evolutionary model “CAT + GTR + G” was selected for all mtDNA datasets using ModelFinder [44]. The BI analyses were carried out using MrBayes ver. 3.2.7 [45], with four independent Markov chains, running for 1,000,000 (complete mitogenomic dataset) and 100,000 (dual and triple concatenated PCGs and individual PCG datasets) metropolises coupled with Monte Carlo generations, sampling a tree every 0.1% generations. The first 25% of the trees were eliminated as “burn-in” when the average standard deviation (SD) of the split frequencies decreased to < 0.01, and the remaining trees were used to calculate Bayesian posterior probabilities (PPs). The evolutionary distance was estimated using the MrBayes order (aamodelpr = mixed) with default parameters. A consensus tree was obtained and visualized using TreeviewX (https://www.linuxlinks.com/treeviewx/).

## Results and discussion

### General features of 33 *Eimeria* mitogenomes

The complete mitogenomes of 33 eimerian isolates representing all seven species known to infect chickens in China varied from 6148 to 6480 bp in size (GenBank accession nos. OP800493–OP800525; Table 1). Each genome encoded three mt PCGs including *cox*1, *cox*3 and *cyt*b as well as seven fragmented small subunit (SSU) and 12 fragmented LSU rRNAs, except for *E. mitis* isolates, which contained another 56-bp LSU rRNA (LSU15) located between LSU13 and LSUD (Fig. 1a–g). The overall base composition of each mitogenome consistently exhibited high AT bias (64.52–67.37%) with T being the most favored base and G the least favored, similar to those observed in other *Eimeria* members [46–50].

**Table 1:** List of Chinese *Eimeria* isolates sequenced in the present study

*Eimeria* isolate	Location	Targeted species	GenBank nos.	Mitogenome size (bp)
Isolate 1	Sichuan	*Eimeria tenella*	OP800493	6213
Isolate 2	Anhui	*Eimeria tenella*	OP800494	6213
Isolate 3	Anhui	*Eimeria tenella*	OP800495	6213
Isolate 4	Sichuan	*Eimeria tenella*	OP800496	6213
Isolate 5	Sichuan	*Eimeria tenella*	OP800497	6213
Isolate 6	Sichuan	*Eimeria tenella*	OP800498	6213
Isolate 7	Anhui	*Eimeria tenella*	OP800499	6213
Isolate 8	Sichuan	*Eimeria necatrix*	OP800500	6212
Isolate 9	Chongqing	*Eimeria necatrix*	OP800501	6212
Isolate 10	Chongqing	*Eimeria necatrix*	OP800502	6212
Isolate 11	Chongqing	*Eimeria necatrix*	OP800503	6212
Isolate 12	Sichuan	*Eimeria necatrix*	OP800504	6212
Isolate 13	Guangdong	*Eimeria necatrix*	OP800505	6212
Isolate 14	Chongqing	*Eimeria maxima*	OP800506	6169
Isolate 15	Guangdong	*Eimeria maxima*	OP800507	6169
Isolate 16	Henan	*Eimeria maxima*	OP800508	6169
Isolate 17	Henan	*Eimeria maxima*	OP800509	6169
Isolate 18	Fujian	*Eimeria maxima*	OP800510	6169
Isolate 19	Anhui	*Eimeria maxima*	OP800511	6169
Isolate 20	Zhejiang	*Eimeria praecox*	OP800512	6174
Isolate 21	Henan	*Eimeria praecox*	OP800513	6174
Isolate 22	Guangdong	*Eimeria praecox*	OP800514	6173
Isolate 23	Chongqing	*Eimeria praecox*	OP800515	6172
Isolate 24	Chongqing	*Eimeria mitis*	OP800516	6408
Isolate 25	Zhejiang	*Eimeria mitis*	OP800517	6408
Isolate 26	Sichuan	*Eimeria mitis*	OP800518	6408
Isolate 27	Fujian	*Eimeria brunetti*	OP800519	6148
Isolate 28	Anhui	*Eimeria brunetti*	OP800520	6156
Isolate 29	Chongqing	*Eimeria brunetti*	OP800521	6156
Isolate 30	Chongqing	*Eimeria brunetti*	OP800522	6157
Isolate 31	Fujian	*Eimeria acervulina*	OP800523	6179
Isolate 32	Fujian	*Eimeria acervulina*	OP800524	6179
Isolate 33	Henan	*Eimeria acervulina*	OP800525	6179

Within SSU rRNAs, seven gene fragments including SSUA, SSUF, SSUD, SSU9, SSU8, SSUB and SSUE ranged from 37 (SSUF) to 116 bp (SSUB) in size. For LSU rRNAs, 12 gene fragments including LSUF, LUSG, LUSC, LSU10, LSU13, LSUD, LSU2, LSUA, LSU1, LUSB, LSU3 and LSUE ranged from 16 (LUSC) to 188 bp (LSUE) in size, very similar to those of other *Eimeria* species reported to date [25, 46–50]. Furthermore, AT-skew and GC-skew values of rRNAs are shown in Fig. 1h, and it was clear that these mitogenomes shared a same skewness pattern: a slightly higher A than T (except for LSU rRNAs) in *E. necatrix* (− 0.003), *E. maxima* (− 0.021) and *E. brunetti* (− 0.008) and a slightly lower G than C (except for SSU rRNAs) in *E. necatrix* (− 0.021) and *E. tenella* (− 0.029).

**Fig. 2:**
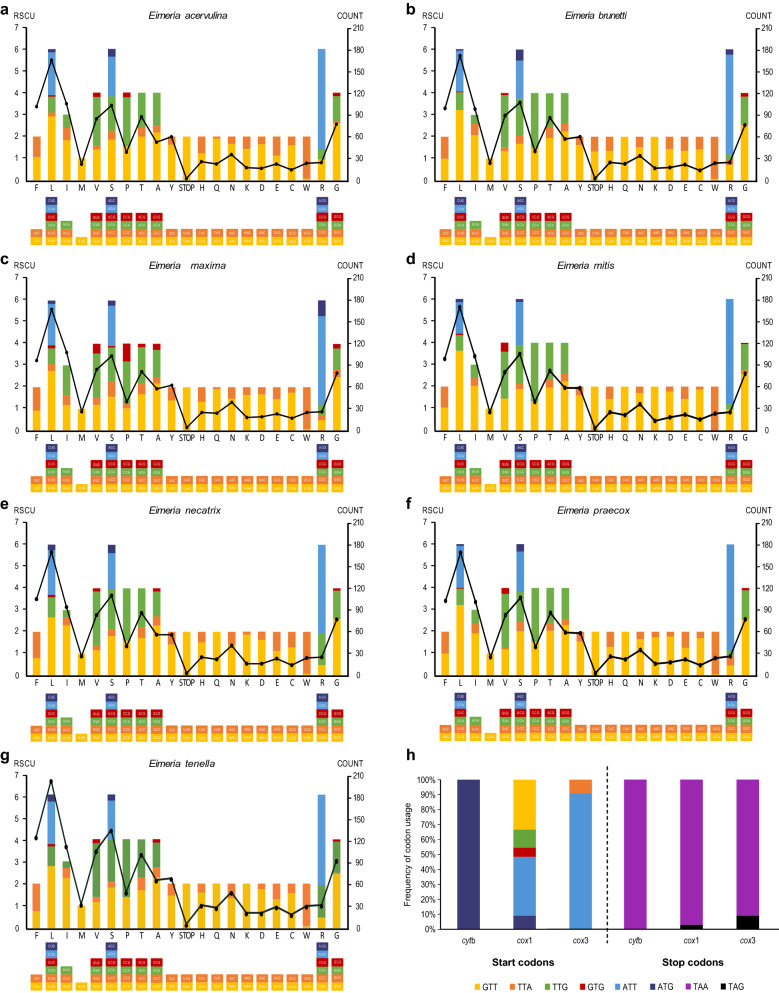
Codon usage in PCGs of mitogenomes of 33 chicken *Eimeria* isolates studied here at the species level. **a–g** RSCU and the number of codons of PCG genes used in each species based on their corresponding isolates. Codon families are plotted under the X axis and represented by different color bars, and the codon numbers are indicated by the black line graph (right axis scale). **h** Start and stop codon usages of three PCG genes in all isolate mitogenomes

For three PCGs, *cox*1, *cox*3 and *cyt*b located between *cyt*b and LSUF, LSUA and LSU1 and SSUA and *cox*1, respectively, with an obvious bias towards AT (Fig. 1). Such AT bias was also reflected in their RSCU and codon usage patterns of PCGs (Fig. 2); for instance, within the initiation codon choice, the *cox*1 gene was inferred to start with codons TTG, ATN (ATT and ATG) and GTN (GTG and GTT), the *cox*3 gene was inferred to start with codons ATT and TTA, and the *cyt*b gene was inferred to start with codon ATG (Fig. 2h). Correspondingly, the standard stop codon TAG was used to terminate the *cox*1 and *cox*3 genes and the *cyt*b gene used TAA as the stop codon, consistent with the previous study [49]. Moreover, RSCU comparisons showed that these 33 eimerian isolates consistently used TAA as the stop codon, except for *E. acervulina* isolates, which used TGA and TAA as stop codons (Fig. 2), inconsistent with mitogenomes of some other Apicomplexa parasites in which an abbreviated stop codon (TA or T) was used as the stop codon [50]. It was also evident that the most frequently used codon of these 33 eimerian isolates was AGA (RSCU ranging from 4.08–4.86), followed by UUA (2.67–3.65), GGU (2.44–2.62) and CCA (1.95–2.67). These codon patterns were similarly reflected in the amino acid usage frequency of PCGs, and the most frequently used amino acid was Leu (count ranging from 166–171), followed by Ser (102–112), Phe (98–104) and Ile (93–108) (Fig. 2a-g).

### Mitogenome diversity

Most gene sizes of mitogenomes of these 33 *Eimeria* isolates were consistent with those of other congeneric species (Additional file 3: Table S3). Nevertheless, to further understand the evolutionary divergence among the genus *Eimeria*, the intra- and interspecific variability was determined across the 33 isolates using the nucleotide and amino acid sequences. The interspecific divergences were determined to range from 1.5 to 12.8% while intraspecific differences were < 0.3% (Additional file 4: Table S4). Both ranges were lower than those estimated in Australian chicken *Eimeria* by Morgan and Godwin [25], to some extent supporting a more general systematic congruence among Chinese chicken eimerian parasites sequenced in this study. Meanwhile, it seemed that the lowest interspecific divergence always occurred between *E. tenella* and *E. necatrix*, suggesting their closer genetic similarity among *Eimeria* species. Furthermore, divergences were also focused on various portions of the mitogenomes. For rRNAs, the nucleotide sequence divergences of SSU and LSU rRNAs ranged from 0.2 to 4.6% and 0.4 to 8.2%, respectively; within PCG genes, the nucleotide sequence divergences ranged from 2.1 to 17.6%, and amino acid sequence divergences ranged from 0.8 to 10.1%, with *cox*3 being the most variable gene, in line with previous findings in species of *Plasmodium*, *Theileria* and *Babesia* [51, 52].

**Fig. 3:**
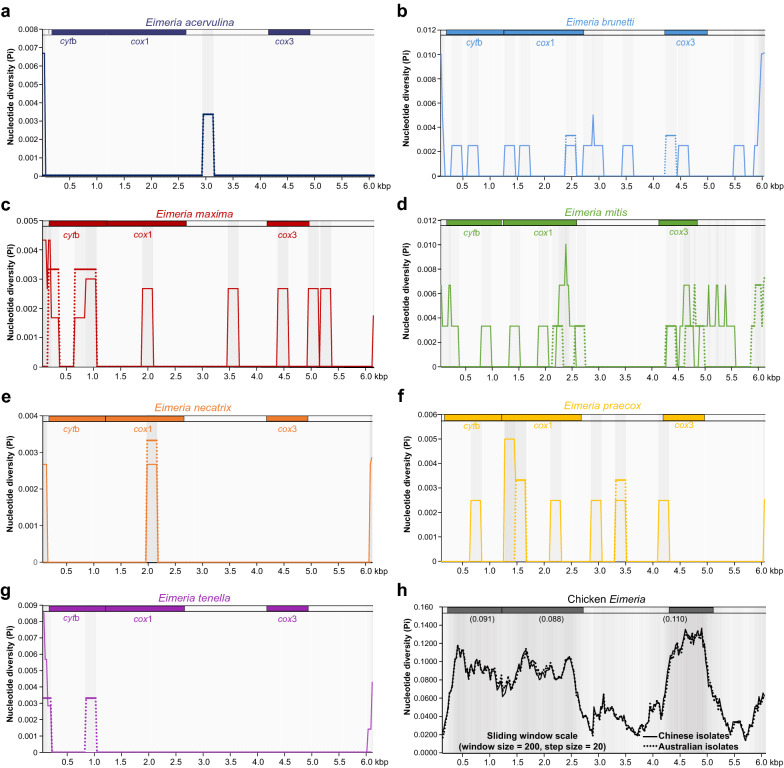
Sliding window analysis of mitogenomes for the nucleotide diversity (Pi) among chicken *Eimeria*. X-axis: position of the midpoint of a window, Y-axis: nucleotide diversity (Pi) of each window (window size: 200 bp; step size: 20 bp). PCG gene boundaries are indicated above the graph. Colors from gray (high diversity) to white (low diversity) indicate the different nucleotide diversities. **a–g** Sliding window analysis of each chicken *Eimeria* species including isolates sequenced in this study. **h** Sliding window analyses of all chicken *Eimeria* species including the 33 isolates sequenced in this study. The Pi value of each PCG is shown near the gene name

To confirm the aforementioned sequence diversities within and between mt genes, sliding window analysis was also carried out by a combination of the 33 *Eimeria* isolates and other congeneric species, with a comparison between Chinese and Australian *Eimeria* populations. As shown in Fig. 3a–g, the intraspecific Pi values of seven *Eimeria* species, regardless of sources (China or Australia), all were relatively low (0 to 0.069) with a significantly increased trend in the following order: *E. acervulina* < *E. tenella* < *E. necatrix* < *E. praecox* < *E. maxima* < *E. brunetti* < *E. mitis*. It was significant that various numbers of peaks presented within comparisons of interspecific diversity depending on species pairs, suggesting that there is still a considerable number of alternative genetic loci to be determined as species-specific markers for identification and differentiation of chicken coccidian parasites. Current mt genetic loci used for PCR detection/diagnostics in the genus *Eimeria* include 805-bp *cox*1 and 954-bp *cox*3 [23, 53], and both genetic loci have been recently targeted for development of an amplicon library-based NGS pipeline analysis for diagnosis [13, 23]. From the analysis in this study, however, compared to the *cox*1 and *cox*3 genes, it appeared that the *cyt*b gene could also be suitable as a genetic marker for diagnosis and identification between *E. maxima*/*E. tenella* and other congeneric species because of its higher variability (Fig. 3c and g). Furthermore, when the sliding window analysis was performed across all chicken *Eimeria* species, the PCG regions with pronounced peaks and troughs seemed to be more significant than other portions of the mitogenomes (Fig. 3h). Among these PCG regions, *cox*1 was determined to be the most conserved while *cox*3 was deemed to be the least conserved, supporting the aforementioned by sequence divergence analysis.

**Fig. 4:**
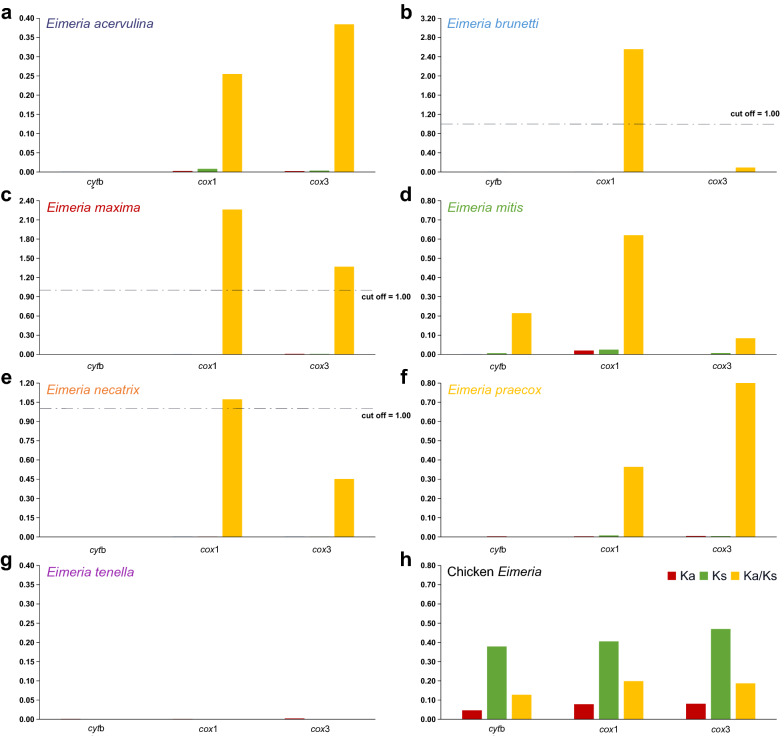
Evolutionary rates of chicken *Eimeria* including the 33 isolates identified in this study. Rate of non-synonymous substitutions (Ka), rate of synonymous substitutions (Ks) and ratio of rate of non-synonymous substitutions to rate of synonymous substitutions (Ka/Ks) are calculated for each PCG. **a–g** Average Ka, Ks and Ka/Ks ratios of three PCGs of isolates reported and sequenced in this study in each species. **h** Average Ka, Ks and Ka/Ks ratios of 66 *Eimeria* isolates including the 33 isolates sequenced in this study

In parallel with the sliding window analysis, the F_ST_ values obtained from PCGs of these *Eimeria* spp. according to their geographical sources (China vs. Australia) are shown in Additional file 5: Table S5. *F*_ST_ of 0–0.18 suggested species-specific variation in levels of interbreeding, with a higher level of genetic isolation between Chinese and Australian *E. mitis* populations. In addition, the ratio of non-synonymous (Ka) and synonymous (Ks) substitutions for each PCG in 33 *Eimeria* isolates and other congeneric species is shown in Fig. 4a–g. Notably, the Ka, Ks and Ka/Ks tied well with results of sliding window analysis, and the PCGs of the *Eimeria* species with high Pi values trended to have positive selection sites (Ka/Ks > 1). For example, Ka/Ks ratios of the *cox*1 gene among *E. brunetti* and *E. necatrix* isolates and the *cox*1 and *cox*3 genes among *E. maxima* isolates all were > 1. In contrast, the Ka/Ks ratios of the *cyt*b gene in all isolates were observed nearby zero, suggesting its strong purifying selection. Moreover, when the Ka/Ks was calculated across all *Eimeria* species, all PCG genes exhibited low ratios (< 1) [54], suggesting that these genes were evolving under negative or purifying selection and, to certain extent, implied the conservation and validity of seven *Eimeria* species infecting chickens.

### Genetic distances of chicken *Eimeria*

**Fig. 5:**
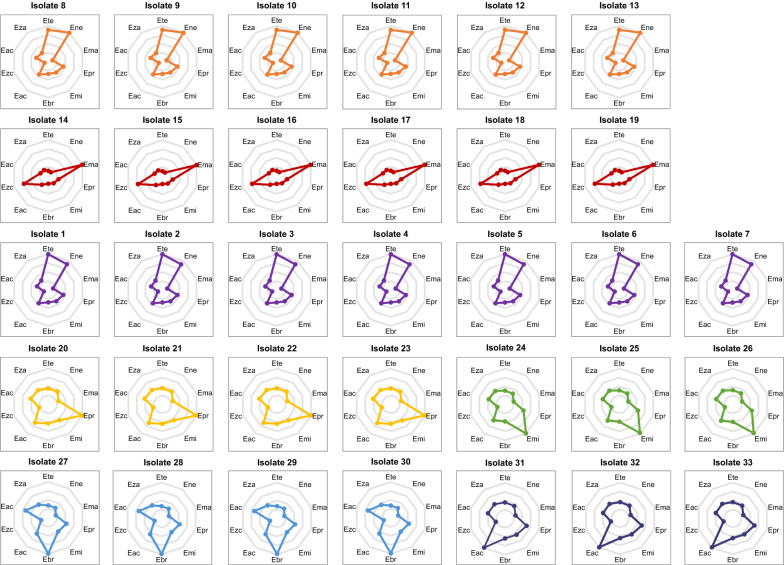
Patterns of K2P distance between 33 isolates and 10 known chicken *Eimeria* species. The edge of these decagons indicates the K2P distance between the isolate and its corresponding species (0.000). Gray lines indicate the same K2P distance from the center of these decagons. Different color dots show the relative K2P distances between isolates with ten *Eimeria* species. The abbreviations Eac, Ebr, Ela, Ema, Emi, Ena, Ene, Epr, Ete and Eza represent *Eimeria acervulina*, *E. brunetti*, *E. lata*, *E. maxima*, *E. mitis*, *E. nagambie*, *E. necatrix*, *E. praecox*, *E. tenella* and *E. zaria*, respectively

We calculated the intraspecific and interspecific genetic distances between these 33 isolates and each of ten chicken eimerian species using the concatenated PCGs. As shown in Fig. 5, the K2P model-based genetic distances between *Eimeria* isolates 1–7 and *E. tenella*, *Eimeria* isolates 8–13 and *E. necatrix*, *Eimeria* isolates 14–19 and *E. maxima*, *Eimeria* isolates 20–23 and *E. praecox*, *Eimeria* isolates 24–26 and *E. mitis*, *Eimeria* isolates 27–30 and *E. brunetti* and *Eimeria* isolates 31–33 and *E. acervulina* approached zero and suggested their species identity, in agreement with the morphological and molecular identifications. For all species clusters, the intraspecific variations were lower than those calculated on the basis of the 18S gene (0.000–0.002 vs. 0.002–0.013) [5]. Moreover, the interspecific genetic distance between *E. tenella* and *E. necatrix* was lower than each compared to the other five *Eimeria* species, once again suggesting that these two species had the closest relationship among chicken eimerian parasites [5, 6, 25, 46–50, 53]. By contrast, the genetic distance between *E. maxima* and other congeneric species appeared the farthest (0.138 to 0.176), consistent with the previous finding [53], supporting a distinct relationship between *E. maxima* and other chicken *Eimeria* species.

### Phylogenies of chicken *Eimeria*

**Fig. 6:**
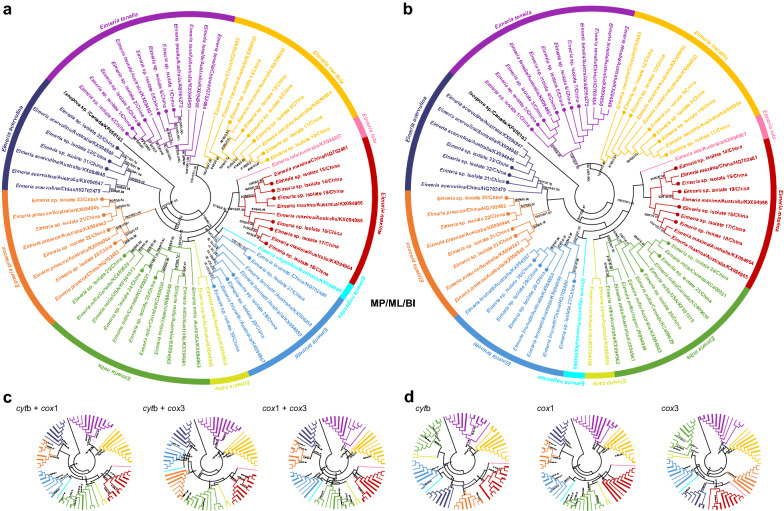
Phylogenetic relationships of chicken *Eimeria* including the 33 isolates identified in this study. **a** Phylogenies were inferred on the basis of the complete mitogenome datasets. **b** Phylogenies were inferred on the basis of the concatenated nucleotide sequences of three PCGs. **c** Phylogenies were inferred on the basis of the concatenated nucleotide sequences of two PCGs. **d** Phylogenies were inferred on the basis of the nucleotide sequences of single PCG. 66 chicken *Eimeria* and one *Isospora* speecies (outgroup) were included in the aforementioned phylogenetic analyses using the BI, ML and MP methods. Ten chicken *Eimeria* species, *E. acervulina*, *E. brunetti*, *E. lata*, *E. maxima*, *E. mitis*, *E. nagambie*, *E. necatrix*, *E. praecox*, *E. tenella* and *E. zaria*, and their branches are shown in dark blue, blue, pink, red, green, cyan, yellow, orange, purple and flavogreen, respectively; 33 *Eimeria* isolates sequenced in this study are indicated in bold font (**a** and **b**) or branches (**c** and **d**). The numbers along the branches indicate bootstrap values/posterior probabilities resulting from different analyses in the order MP/ML/BI

The available mitogenomes of 33 isolates representing all seven known chicken eimerian species in China together with an additional 25 mitogenomes of Australian *Eimeria* spp. provided us an opportunity to study the evolutionary relationships of each isolate in each species and of each species in the genus *Eimeria*. As shown in Fig. 6a and b, it was clear that three identical trees (MP/ML/BI) inferred from either the complete mitogenomes or concatenated PCG datasets consistently revealed that the *Eimeria* isolates 1–7 were grouped with available species of *E. tenella*, the *Eimeria* isolates 8–13 were grouped with *E. necatrix*, the *Eimeria* isolates 14–19 were grouped with *E. maxima*, the *Eimeria* isolates 20–23 were grouped with *E. praecox*, *Eimeria* isolates 24–26 were grouped with *E. mitis*, *Eimeria* isolates 27–30 were grouped with *E. brunetti* isolates, and *Eimeria* isolates 31–33 were grouped with *E. acervulina*, with high statistical supports (all values ≥ 98 or = 1.00). Furthermore, both phylogenetic trees split chicken eimerian parasites into two clades: one included *E. tenella* and *E. necatrix* and another was composed of *E. lata*, *E. maxima*, *E. nagambie*, *E. brunetti*, *E. zaria*, *E. mitis*, *E. praecox* and *E. acervulina*. Compared to the stable topology of *E. tenella* and *E. necatrix*, the phylogenetic relationships among eight other *Eimeria* species varied by different datasets used here (Fig. 6a and b). Notably, *E. maxima* and *E. mitis* were more closely related to each other than to *E. praecox* and *E. acervulina*, in contrast with findings based on the nuclear 18S and internal transcribed spacer (ITS) and mitochondrial *cox*1 datasets [53, 55–57]. Nevertheless, these analyses provided a consistent, robust phylogenetic resolution for the 33 isolates and their congeneric species in the genus *Eimeria*: a paraphyletic relationship was shared among the 10 chicken *Eimeria* species, in agreement with our genetic distance study herein and the results of morphological and molecular biology studies [6, 56, 58–60].

Furthermore, the single and concatenated PCG genes were separately used for phylogenetic analysis to screen out the optimal genetic marker candidates for phylogeny and species identification and differentiation of chicken eimerian parasites from other related species. As shown in Fig. 6c and d, although any single or dual combination of PCGs exhibited various phylogenetic topologies, the positions of the species *E. tenella* and *E. necatrix* were steady in these six (*cox*1, *cox*3, *cyt*b, *cyt*b + *cox*1, *cyt*b + *cox*3 and *cox*1 + *cox*3) phylogenetic analyses. Notably, the concatenated *cyt*b and *cox*1 gene- and single *cox*1-based phylogenetic analyses shared the same topology as that of the genome-based phylogeny, suggesting that the *cox*1 gene might be the most appropriate genetic marker and therefore could be used instead of mitogenomes for evolutionary and phylogenetic studies of chicken *Eimeria* species. Of course, the marker validity of the *cox*1 gene remains further validated when more additional apicomplexan parasite mitogenomes become available, especially those from avain coccidia, although the *cox*1 has been widely used as a DNA barcode for species identification and differentiation in *Eimeria* [53, 61].

## Conclusions

In this study, we presented a comprehensive molecular characterization of evolutionary blueprint of chicken coccidia by Illumina sequencing complete mitogenomes of 33 isolates representing all seven known *Eimeria* species infecting chickens in China. Comparative genomics revealed the low genetic diversity of these *Eimeria* species and showed three mitochondrial protein genes under purifying selection with *cox*1 and *cox*3 genes being the lowest and highest evolutionary rates, respectively. Phylogenies divided these 33 *Eimeria* isolates into seven subgroups, and each represented one chicken *Eimeria* species. Furthermore, single and concatenated mitochondrial protein gene-based phylogenies supported the *cox*1 gene as the genetic marker for evolutionary and phylogenetic studies for avain eimerians. These *Eimeria* population-level mitogenomic datasets provide an updated understanding of systematic, population genetic and evolutionary biological studies of apicomplexan parasites in poultry and other animals.

## Supplementary Information

**Additional file 1: Table S1.** List of primer pairs for PCR amplifications and their positions in the mitogenome of *Eimeria* isolate 1.

**Additional file 2: Table S2.** Summary of the chicken *Eimeria* mitogenomic information included in this study.

**Additional file 3: Table S3.** Gene sizes in the mitogenomes of 66 chicken *Eimeria* parasites.

**Additional file 4: Table S4.** Pairwise genetic divergences of 33 chicken *Eimeria* parasites based on mitogenome datasets.

**Additional file 5: Table S5.** Wrights Fixation Index (F_ST_) values of Chinese and Australian chicken *Eimeria* species based on their mitochondrial concatenate PCGs.

## Data Availability

Molecular data have been deposited to GenBank with the following accession numbers: OP800493–OP800525.

## Abbreviations

18S: Nuclear ribosomal protein 18
ITS-1: Internal transcribed spacer 1
ITS-2: Internal transcribed spacer 2
MP: Maximum parsimony
ML: Maximum likelihood
BI: Bayesian inference
PCG: Protein-coding gene
tRNA: Transfer RNA
rRNA: Ribosomal RNA
LSU: Large subunit ribosomal RNA
SSU: Small subunit ribosomal RNA
*cox*1: Cytochrome oxidase subunits 1
*cox*3: Cytochrome oxidase subunits 3
*cyt*b: Cytochrome b
F_ST_: Wright’s fixation index
Ka: Non-synonymous substitutions
Ks: Synonymous substitutions

## References

[1] Fatoba AJ, Adeleke MA (2018). Diagnosis and control of chicken coccidiosis: a recent update. J Parasit Dis.

[2] Soutter F, Werling D, Tomley FM, Blake DP (2020). Poultry coccidiosis: design and interpretation of vaccine studies. Front Vet Sci.

[3] Chapman HD, Barta JR, Blake D, Gruber A, Jenkins M, Smith NC, Suo X, Tomley FM (2013). A selective review of advances in coccidiosis research. Adv Parasitol.

[4] Cantacessi C, Riddell S, Morris GM, Doran T, Woods WG, Otranto D, Gasser RB (2008). Genetic characterization of three unique operational taxonomic units of *Eimeria* from chickens in Australia based on nuclear spacer ribosomal DNA. Vet Parasitol.

[5] Clark EL, Macdonald SE, Thenmozhi V, Kundu K, Garg R, Kumar S, Ayoade S, Fornace KM, Jatau ID, Moftah A, Nolan MJ, Sudhakar NR, Adebambo AO, Lawal IA, Álvarez Zapata R, Awuni JA, Chapman HD, Karimuribo E, Mugasa CM, Namangala B, Rushton J, Suo X, Thangaraj S, Srinivasa Rao ASR, Tewari AK, Banerjee PS, Dhinakar Raj G, Raman M, Tomley FM, Blake DP (2016). Cryptic *Eimeria* genotypes are common across the southern but not northern hemisphere. Int J Parasitol.

[6] Blake DP, Vrba V, Xia D, Jatau ID, Spiro S, Nolan MJ, Underwood G, Tomley FM (2021). Genetic and biological characterisation of three cryptic *Eimeria* operational taxonomic units that infect chickens (*Gallus gallus domesticus*). Int J Parasitol.

[7] Andreopoulou M, Chaligiannis I, Sotiraki S, Daugschies A, Bangoura B (2022). Prevalence and molecular detection of *Eimeria* species in different types of poultry in Greece and associated risk factors. Parasitol Res.

[8] Gottardo Balestrin PW, Balestrin E, Santiani F, Biezus G, Moraes JC, da Silva Casa M, Medeiros ALV, Casagrande RA (2021). Prevalence of *Eimeria* sp. in broiler poultry houses with positive and negative pressure ventilation systems in southern Brazil. Avian Dis.

[9] Karaer Z, Guven E, Akcay A, Kar S, Nalbantoglu S, Cakmak A (2012). Prevalence of subclinical coccidiosis in broiler farms in Turkey. Trop Anim Health Prod.

[10] Kumar S, Garg R, Ram H, Maurya PS, Banerjee PS (2015). Gastrointestinal parasitic infections in chickens of upper gangetic plains of India with special reference to poultry coccidiosis. J Parasit Dis.

[11] Györke A, Pop L, Cozma V (2013). Prevalence and distribution of *Eimeria* species in broiler chicken farms of different capacities. Parasite.

[12] Debbou-Iouknane N, Benbarek H, Ayad A (2018). Prevalence and aetiology of coccidiosis in broiler chickens in Bejaia province, Algeria. Onderstepoort J Vet Res.

[13] Hauck R, Carrisosa M, McCrea BA, Dormitorio T, Macklin KS (2019). Evaluation of next-generation amplicon sequencing to identify *Eimeria* spp. of chickens. Avian Dis.

[14] Huang Y, Ruan X, Li L, Zeng M (2017). Prevalence of *Eimeria* species in domestic chickens in Anhui province, China. J Parasit Dis.

[15] Blake DP, Knox J, Dehaeck B, Huntington B, Rathinam T, Ravipati V, Ayoade S, Gilbert W, Adebambo AO, Jatau ID, Raman M, Parker D, Rushton J, Tomley FM (2020). Re-calculating the cost of coccidiosis in chickens. Vet Res.

[16] Coker S. Morphological and molecular characterisation of coccidia (*Eimeria* spp.) in kiwi (*Apteryx* spp.). Animal Science at Massey University, Palmerston North, New Zealand; 2021.

[17] Geng T, Ye C, Lei Z, Shen B, Fang R, Hu M, Zhao J, Yanqin Z (2021). Prevalence of *Eimeria* parasites in the Hubei and Henan provinces of China. Parasitol Res.

[18] Vermeulen ET, Lott MJ, Eldridge MD, Power ML (2016). Evaluation of next generation sequencing for the analysis of *Eimeria* communities in wildlife. J Microbiol Methods.

[19] Hinsu AT, Thakkar JR, Koringa PG, Vrba V, Jakhesara SJ, Psifidi A, Guitian J, Tomley FM, Rank DN, Raman M, Joshi CG, Blake DP (2018). Illumina next generation sequencing for the analysis of *Eimeria* populations in commercial broilers and indigenous chickens. Front Vet Sci.

[20] Barr CM, Neiman M, Taylor DR (2005). Inheritance and recombination of mitochondrial genomes in plants, fungi and animals. New Phytol.

[21] Lin CP, Danforth BN (2004). How do insect nuclear and mitochondrial gene substitution patterns differ? Insights from Bayesian analyses of combined datasets. Mol Phylogenet Evol.

[22] Hao W, Richardson AO, Zheng Y, Palmer JD (2010). Gorgeous mosaic of mitochondrial genes created by horizontal transfer and gene conversion. Proc Natl Acad Sci U S A.

[23] Snyder RP, Guerin MT, Hargis BM, Imai R, Kruth PS, Page G, Rejman E, Barta JR (2021). Exploiting digital droplet PCR and next generation sequencing technologies to determine the relative abundance of individual *Eimeria* species in a DNA sample. Vet Parasitol.

[24] Rokas A, Williams BL, King N, Carroll SB (2003). Genome-scale approaches to resolving incongruence in molecular phylogenies. Nature.

[25] Morgan JAT, Godwin RM (2017). Mitochondrial genomes of Australian chicken *Eimeria* support the presence of ten species with low genetic diversity among strains. Vet Parasitol.

[26] Goan C, Walker F. Poultry litter sampling and testing. In: The university of Tennessee, agricultural extension service. 2009. https://extension.tennessee.edu/publications/Documents/SP563.pdf. Accessed Feb 2018.

[27] Castañón CAB, Fraga JS, Fernandez S, Gruber A, da Costa FL (2007). Biological shape characterization for automatic image recognition and diagnosis of protozoan parasites of the genus *Eimeria*. Pattern Recognit.

[28] Haug A, Gjevre AG, Thebo P, Mattsson JG, Kaldhusdal M (2008). Coccidial infections in commercial broilers: epidemiological aspects and comparison of *Eimeria* species identification by morphometric and polymerase chain reaction techniques. Avian Pathol.

[29] Schwarz RS, Jenkins MC, Klopp S, Miska KB (2009). Genomic analysis of *Eimeria* spp. populations in relation to performance levels of broiler chicken farms in Arkansas and North Carolina. J Parasitol.

[30] Hahn C, Bachmann L, Chevreux B (2013). Reconstructing mitochondrial genomes directly from genomic next-generation sequencing reads—a baiting and iterative mapping approach. Nucleic Acids Res.

[31] Bernt M, Donath A, Jühling F, Externbrink F, Florentz C, Fritzsch G, Pütz J, Middendorf M, Stadler PF (2013). MITOS: improved *de novo* metazoan mitochondrial genome annotation. Mol Phylogenet Evol.

[32] Kearse M, Moir R, Wilson A, Stones-Havas S, Cheung M, Sturrock S, Buxton S, Cooper A, Markowitz S, Duran C, Thierer T, Ashton B, Meintjes P, Drummond A (2012). Geneious basic: an integrated and extendable desktop software platform for the organization and analysis of sequence data. Bioinformatics.

[33] Zhang D, Gao F, Jakovlić I, Zou H, Zhang J, Li WX, Wang GT (2020). PhyloSuite: an integrated and scalable desktop platform for streamlined molecular sequence data management and evolutionary phylogenetics studies. Mol Ecol Resour.

[34] Perna NT, Kocher TD (1995). Patterns of nucleotide composition at fourfold degenerate sites of animal mitochondrial genomes. J Mol Evol.

[35] Burland TG (2000). DNASTAR’s lasergene sequence analysis software. Methods Mol Biol.

[36] Kumar S, Stecher G, Li M, Knyaz C, Tamura K (2018). MEGA X: molecular evolutionary genetics analysis across computing platforms. Mol Biol Evol.

[37] Rozas J, Ferrer-Mata A, Sánchez-DelBarrio JC, Guirao-Rico S, Librado P, Ramos-Onsins SE, Sánchez-Gracia A (2017). DnaSP 6: DNA sequence polymorphism analysis of large data sets. Mol Biol Evol.

[38] Zhang Z, Li J, Zhao XQ, Wang J, Wong GK, Yu J (2006). KaKs_Calculator: calculating Ka and Ks through model selection and model averaging. Genom Proteom Bioinf.

[39] Srivathsan A, Meier R (2012). On the inappropriate use of Kimura-2-parameter (K2P) divergences in the DNA-barcoding literature. Cladistics.

[40] Katoh K, Misawa K, Kuma K, Miyata T (2002). MAFFT: a novel method for rapid multiple sequence alignment based on fast fourier transform. Nucleic Acids Res.

[41] Goloboff PA, Catalano SA, Torres A (2022). Parsimony analysis of phylogenomic datasets (II): evaluation of PAUP*. MEGA and MPBoot Cladistics.

[42] Castresana J (2000). Selection of conserved blocks from multiple alignments for their use in phylogenetic analysis. Mol Biol Evol.

[43] Guindon S, Gascuel O (2003). A simple, fast, and accurate algorithm to estimate large phylogenies by maximum likelihood. Syst Biol.

[44] Kalyaanamoorthy S, Minh BQ, Wong TKF, von Haeseler A, Jermiin LS (2017). ModelFinder: fast model selection for accurate phylogenetic estimates. Nat Methods.

[45] Ronquist F, Teslenko M, van der Mark P, Ayres DL, Darling A, Höhna S, Larget B, Liu L, Suchard MA, Huelsenbeck JP (2012). MrBayes 3.2: efficient bayesian phylogenetic inference and model choice across a large model space. Syst Biol.

[46] Ogedengbe ME, Hafeez MA, Barta JR (2013). Sequencing the complete mitochondrial genome of *Eimeria mitis* strain USDA 50 (Apicomplexa: Eimeriidae) suggests conserved start positions for mtCOI- and mtCOIII-coding regions. Parasitol Res.

[47] Liu G, Li Q, Wang C, Xu C (2019). The complete mitochondrial genome of *Eimeria anseris* from the wintering greater white-fronted goose in Shengjin Lake, China, and phylogenetic relationships among *Eimeria* species. Parasitol Res.

[48] Hafeez MA, Vrba V, Barta JR (2016). The complete mitochondrial genome sequence of *Eimeria innocua* (Eimeriidae, Coccidia, Apicomplexa). Mitochondrial DNA A DNA Mapp Seq Anal.

[49] Liu GH, Hou J, Weng YB, Song HQ, Li S, Yuan ZG, Lin R-G, Zhu X-Q (2012). The complete mitochondrial genome sequence of *Eimeria mitis* (Apicomplexa: Coccidia). Mitochondrial DNA.

[50] Lin RQ, Qiu LL, Liu GH, Wu XY, Weng YB, Xie WQ, Hou J, Pan H, Yuan Z-G, Zou F-C, Hu M, Zhu X-Q (2011). Characterization of the complete mitochondrial genomes of five *Eimeria* species from domestic chickens. Gene.

[51] Perkins SL (2008). Molecular systematics of the three mitochondrial protein-coding genes of malaria parasites: corroborative and new evidence for the origins of human malaria. DNA Seq.

[52] Hikosaka K, Watanabe YI, Tsuji N, Kita K, Kishine H, Arisue N, Palacpac NMQ, Kawazu S-I, Sawai H, Horii T, Igarashi I, Tanabe K (2010). Divergence of the mitochondrial genome structure in the apicomplexan parasites, *Babesia* and *Theileria*. Mol Biol Evol.

[53] Ogedengbe JD, Hanner RH, Barta JR (2011). DNA barcoding identifies *Eimeria* species and contributes to the phylogenetics of coccidian parasites (Eimeriorina, Apicomplexa, Alveolata). Int J Parasitol.

[54] Hurst LD (2002). The Ka/Ks ratio: diagnosing the form of sequence evolution. Trends Genet.

[55] Vrba V, Pakandl M (2015). Host specificity of turkey and chicken *Eimeria*: controlled cross-transmission studies and a phylogenetic view. Vet Parasitol.

[56] Alam MZ, Dey AR, Rony SA, Parvin S, Akter S (2022). Phylogenetic analysis of *Eimeria tenella* isolated from the litter of different chicken farms in Mymensingh, Bangladesh. Vet Med Sci.

[57] Fornace KM, Clark EL, Macdonald SE, Namangala B, Karimuribo E, Awuni JA, Thieme O, Blake DP, Rushton J (2013). Occurrence of *Eimeria* species parasites on small-scale commercial chicken farms in Africa and indication of economic profitability. PLoS ONE.

[58] Barta JR, Martin DS, Liberator PA, Dashkevicz M, Anderson JW, Feighner SD, Elbrecht A, Perkins-Barrow A, Jenkins MC, Danforth HD, Ruff MD, Profous-Juchelka H (1997). Phylogenetic relationships among eight *Eimeria* species infecting domestic fowl inferred using complete small subunit ribosomal DNA sequences. J Parasitol.

[59] Miska KB, Schwarz RS, Jenkins MC, Rathinam T, Chapman HD (2010). Molecular characterization and phylogenetic analysis of *Eimeria* from turkeys and gamebirds: implications for evolutionary relationships in Galliform birds. J Parasitol.

[60] Hafeez MA, Sattar A, Khalid K, Khalid AR, Mahmood MS, Aleem MT, Ashraf K, Aslam F, Alouffi A, Mohammed A, Almutairi MM, Haq MIU (2022). Molecular and morphological characterization of *Eimeria crandallis* isolated from deer (*Cervidae*) in different captive animals. Life (Basel)..

[61] Hafeez MA, Shivaramaiah S, Dorsey KM, Ogedengbe ME, El-Sherry S, Whale J, Cobean J, Barta JB (2015). Simultaneous identification and DNA barcoding of six *Eimeria* species infecting turkeys using PCR primers targeting the mitochondrial cytochrome c oxidase subunit I (mtCOI) locus. Parasitol Res.

